# The feasibility and reliability of measuring forearm muscle thickness by ultrasound in a geriatric inpatient setting: a cross-sectional pilot study

**DOI:** 10.1186/s12877-022-02811-3

**Published:** 2022-02-18

**Authors:** Delky Meza-Valderrama, Dolores Sánchez- Rodríguez, Stany Perkisas, Xavi Duran, Sophie Bastijns, Vanesa Dávalos-Yerovi, Elizabeth Da Costa, Ester Marco

**Affiliations:** 1 Rehabilitation Research Group, Hospital Del Mar Medical Research Institute (IMIM), Carrer de Llull, 410, 08019 Barcelona, Catalonia Spain; 2 Physical Medicine and Rehabilitation Department, National Institute of Physical Medicine and Rehabilitation (INMFRE), Panama City, Panama; 3 Physical Medicine and Rehabilitation Department, Caja de Seguro Social (C.S.S.), Panama City, Panama; 4 Geriatrics Department, Brugmann University Hospital, Université Libre de Bruxelles, Brussels, Belgium; 5 WHO Collaborating Centre for Public Health Aspects of Musculo‑Skeletal Health and Aging, Division of Public Health, Epidemiology and Health Economics, University of Liège, Liège, Belgium; 6 University Center of Geriatrics, Antwerp University, Leopoldstraat 26, 2000 Antwerp, Belgium; 7 First Line and Interdisciplinary Care Medicine, ELIZA, University of Antwerp, 2650 Edegem, Belgium; 8 Methodological and Biostatistical Advisory Service. Hospital del Mar Research Institute, Barcelona, Catalonia Spain; 9 Physical Medicine and Rehabilitation Department, Vall d’Hebron University Hospital, Catalonia Barcelona, Spain; 10 Physical Medicine and Rehabilitation Department, Hospital Del Mar - Hospital de L´Esperança, Parc de Salut Mar, Barcelona, Catalonia Spain; 11 Geriatric Department, Centre Fòrum-Hospital del Mar, Parc de Salut Mar, Barcelona, Catalonia Spain; 12 School of Medicine, Universitat Autònoma de Barcelona, Barcelona, Catalonia Spain; 13 School of Medicine, Universitat Internacional de Catalunya, Sant Cugat del Vallès, Barcelona, Catalonia Spain

**Keywords:** Muscle ultrasound, Forearm muscle thickness, Intra-rater reliability, Inter-rater reliability, Ultrasound feasibility

## Abstract

**Background:**

Given the potential benefits of introducing ultrasound in the clinical assessment of muscle disorders, this study aimed to assess the feasibility and reliability of measuring forearm muscle thickness by ultrasound in a geriatric clinical setting.

**Methods:**

Cross-sectional pilot study in 25 participants (12 patients aged ≥ 70 years in an acute geriatric ward and 13 healthy volunteers aged 25–50 years), assessed by three raters. Muscle thickness measurement was estimated as the distance between the subcutaneous adipose tissue-muscle interface and muscle-bone interface of the radius at 30% proximal of the distance between the styloid process and distal insertion of the *biceps brachii* muscle of the dominant forearm. Examinations were repeated three times by each rater and intra- and inter-rater reliability was calculated. Feasibility analysis included consideration of technological, economic, legal, operational, and scheduling (TELOS) components.

**Results:**

Mean muscle-thickness measurement difference between groups was 4.4 mm (95% confidence interval [CI] 2.4 mm to 6.3 mm], *p* < 0.001). Intra-rater reliability of muscle-thickness assessment was excellent, with intraclass correlation coefficient (ICC) of 0.947 (95%CI 0.902 to 0.974), 0.969 (95%CI 0.942 to 0.985), and 0.950 (95%CI 0.907 to 0.975) for observer A, B, and C, respectively. Inter-rater comparison showed good agreement (ICC of 0.873 [95%CI 0.73 to 0.94]). Four of the 17 TELOS components considered led to specific recommendations to improve the procedure’s feasibility in clinical practice.

**Conclusion:**

Our findings suggest that US is a feasible tool to assess the thickness of the forearm muscles with good inter-rater and excellent intra-rater reliability in a sample of hospitalized geriatric patients, making it a promising option for use in clinical practice.

## Background

Sarcopenia is a progressive and generalized muscle disease associated with increased likelihood of adverse outcomes [1]. According to the 2018 updated definition from the European Working Group of Sarcopenia on Older People (EGWSOP2), sarcopenia is suspected in presence of low muscle strength and confirmed by documentation of low muscle quantity or quality [2].

The EWGSOP2 cut-off points to define reduced muscle quantity are based on dual-energy X-ray absorptiometry (DEXA) and bio-impedance analysis (BIA) (2). Based on feasibility, accuracy, and low cost, DEXA has been considered as the reference standard for measuring muscle mass in patients with sarcopenia [3]. In light of the latest findings in sarcopenia research, the use of ultrasound (US) –a technique largely known for its diagnostic properties in muscle assessment– has been revisited as a promising tool to measure muscle quantity and quality, both of which are technically difficult to assess. Studies using this portable and inexpensive method that does not use ionizing radiation [4] have shown a strong positive correlation with DEXA [5–8], computerized tomography [9], and magnetic resonance imaging (MRI) results [10–12].

Muscle US has good intra- and inter-rater reliability, as well as test–retest reliability in both older adults and younger populations [13, 14]; nevertheless, more research is needed, especially in the US measures of muscle size in small muscles [13] and clinical populations [14]. The SARCopenia measurement by UltraSound (SARCUS) project, a European collaborative partnership, proposes various measurements, including muscle thickness as among the most commonly used to evaluate muscle mass in the upper and lower limbs [4, 15]. Lack of expertise in US among physicians is one of the critical limitations for its implementation in clinical practice. However, published research shows that a brief training course achieves improvement in point-of-care US image interpretation skills and confidence [16, 17] and a reliable endpoint can be achieved by the practitioner who is inexperienced in more complex musculoskeletal US techniques [18].

The rectus femoris and lateral vastus muscles are the most often studied with US in sarcopenia research [4]. However, some degree of patient collaboration (need to undress) and observer effort (assistance to position the patient on the examining table or bed, time required for the protocol) is involved. For this reason, our study proposed a forearm muscle evaluation protocol with the patient seated naturally and the forearm in a neutral position accessible for US transducer placement. This pilot study aimed to assess the feasibility of assessing thickness of the forearm muscles by US and to calculate intra- and inter-rater reliability of these measurements performed by inexperienced users of the technique in a geriatric inpatient setting.

## Methods

### Study design and setting

This was a cross-sectional pilot study reported according to the Strengthening the Reporting of Observational Studies in Epidemiology (STROBE) recommendations [19]. The study was performed in the geriatric ward of the University Center of Geriatrics in Antwerp, Belgium in June 2019.

### Participants

Volunteers were recruited from two different age groups and settings: 12 patients aged 70 years or older (6 women and 6 men, aged 81.8 ± 4.2 years) admitted in an acute geriatric ward who agreed to participate and were able to hold their forearm in the needed position for evaluation, and 13 healthy adult volunteers (doctors, nurses, and social workers at the study site) younger than 50 years (11 women and 2 men, aged 32.9 ± 8.3 years), without known pathologies. Patients with recent surgery or trauma (< 3 months) or neurologic diseases affecting the dominant arm were excluded.

### Variables

**Fig. 1:**
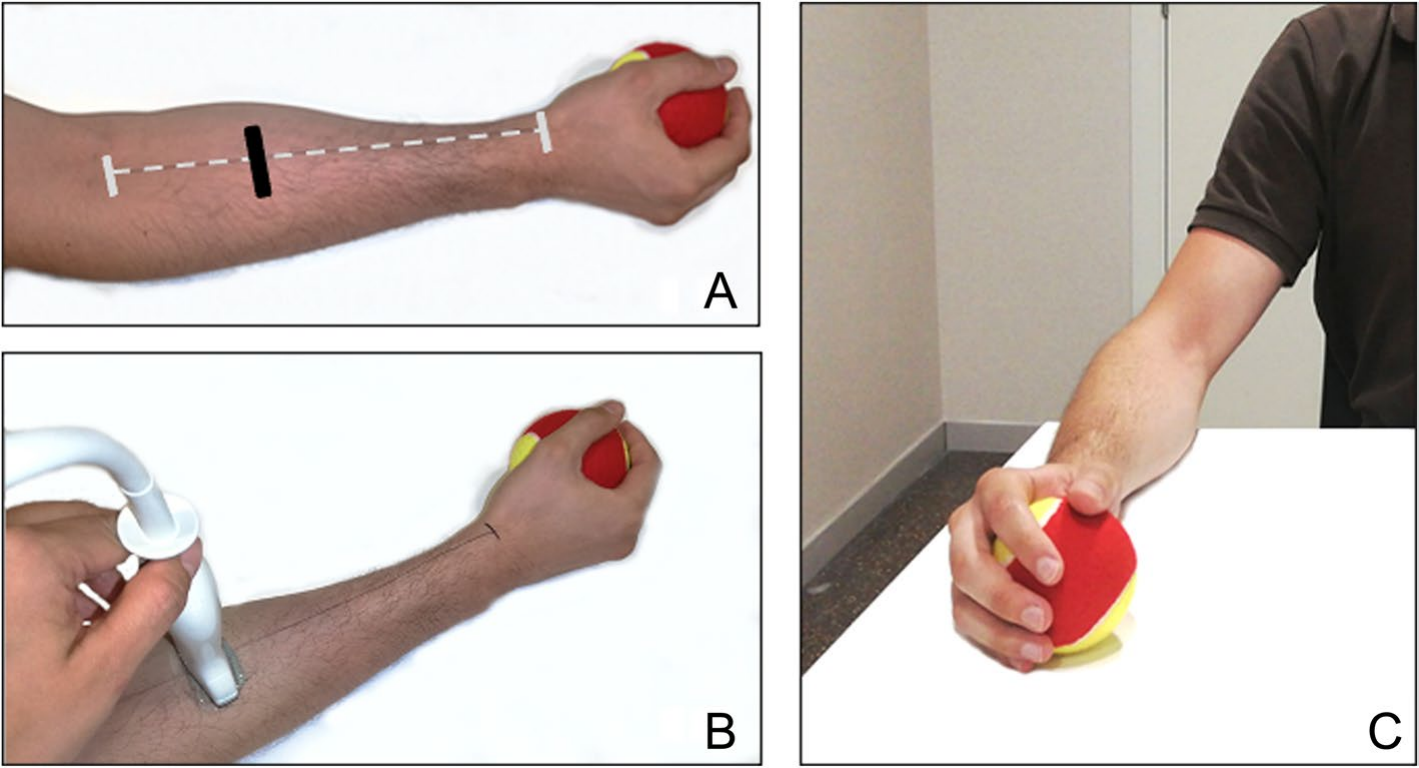
Measurement of muscle thickness, Legend: **A**, Muscle thickness was measured at the lateral dominant forearm at 30% proximal of the distance between the styloid process and the distal insertion of the *biceps brachii* muscle into the radial tuberosity. The black mark indicates where the images should be captured. **B**, A 5 cm width, 7.5 MHz linear transducer was placed perpendicular to the length of the arm. **C**, The patient must be seated with the dominant arm resting on a standard table with shoulders flexed to 30-45º, elbow to 135º, and forearm in neutral position

**Fig. 2:**
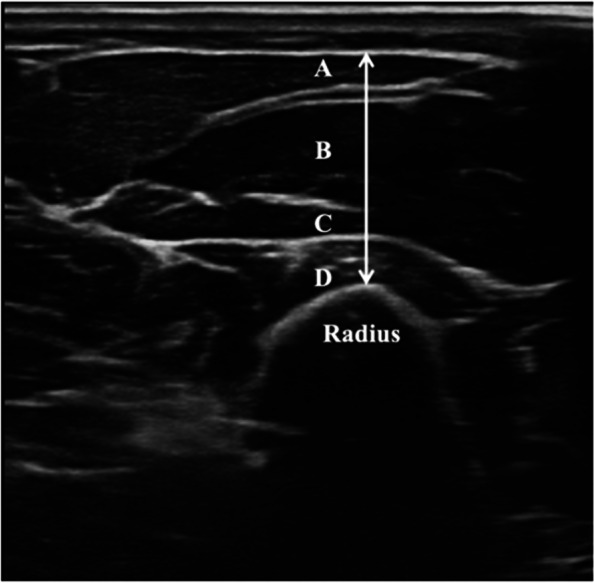
Cross-sectional sonogram of a healthy volunteer, Legend: Forearm muscles assessed: **A**, Brachioradialis muscle. **B**, Extensor carpi radialis longus muscle. **C**, Extensor carpi radialis brevis muscle. **D**, Supinator muscle

- To determine muscle thickness of the forearm**,** each researcher measured the forearm and marked the point of transducer placement on the lateral right forearm, 30% proximal of the distance between the styloid process of the radius and the insertion of the biceps brachii muscle into the radial tuberosity (Fig. 1A). Using B-mode on the Aplio 300 (Canon Medical Systems Europe B.V.), a 5-cm wide 7.5-MHz linear transducer with a scanning head coated with water-soluble transmission gel was used; the probe was placed perpendicular to the length of the arm; minimal pressure was maintained between the transducer and the skin (Fig. 1B). The examination was performed with the patient seated in a chair without armrests, with feet fully resting on the floor, and hips and knees positioned at approximately 90°. The upper limb was resting on a table and positioned as follows: 30-45º shoulder flexion, 45º elbow flexion, mid pronation/supination, 15–30° wrist extension and 0–15° ulnar deviation. To facilitate standardization of positioning, a tennis ball (Wilson Sporting Goods Company, United States of America) was gently held in the hand (Fig. 1C); this position helps to counteract any volitional or nonvolitional contractions of the musculature to hold the forearm as directed. Once the image was selected, the distance between the subcutaneous adipose tissue-muscle interface and the radius muscle-bone interface was measured (Fig. 2). The mean value of three reproducible measurements (< 15% variability) was used for analysis. Each researcher positioned the patient, measured and marked the transducer position in the forearm, captured the image, measured the muscle thickness, and removed any skin marks before the next investigator entered the examination room. A specialist in Geriatrics with US expertise (2 years working experience) provided a 45-min training session; the assessments were carried out by three researchers (raters A, B, and C) with limited or no previous experience in muscle US.
- Feasibility of the procedure was assessed with technological, economic, legal, operational, and scheduling (TELOS) components, adapted from previous studies [20]. For the purpose of this study, 17 yes/no questions (outlined below) with their expected answers were agreed. The components were considered feasible if the answers were those expected; otherwise, actions to resolve barriers were described. Additional feasibility considerations included time spent on the examination, patient discomfort during the procedure, and occurrence of adverse or unexpected events.

### Training of evaluators

Before starting data collection, a specialist in Geriatrics with US expertise (2 years working experience) provided a 45-min training session designed for physicians with limited or no previous experience in muscle US. The training consisted of three parts:

1) Theory: Content included a) generalities in using the US scanner (including adequate grip of the US-transducer, the quantity of gel needed, and pressure management over the area to be evaluated; b) visualization, capture, and measurement of the US image; and c) the patient's position and the measurement of the anatomical point on the forearm for the evaluation.

2) Practical training: The expert measured the forearm muscle thickness by US in a healthy volunteer; each participant then performed the measurement in healthy volunteers under expert supervision.

3) Evaluation: At the end of the training, the expert verified that researchers had completed the entire forearm muscle thickness measurement independently and adequately. Finally, all three researchers (raters A, B, and C) carried out the assessments for this research.

### Study size

A sample size of at least 24 observations was determined, taking into account a scenario with three raters, 5% significance level, and accepting an alpha risk of 0.05 and a beta risk of 0.2 in a two-sided test. We used the formula provided by Zou [21] using the R Package “ICC.Sample.Size” [22], giving a 0.9 hypothesized value of the intraclass correlation coefficient and a null hypothesis value of 0.75.

### Statistical analysis

Categorical variables are described with absolute values and percentages, quantitative variables with mean and standard deviation (SD). The assumption of normality of the quantitative variables was checked with normal probability graphs and the Kolmogorov–Smirnov test corrected by the Lilliefors test. Student t-test for independent samples was used to assess age-related differences in muscle-thickness. The intraclass correlation coefficient (ICC) and its 95% confidence intervals (CI) were calculated to determine both the intra- and inter-rater agreement [23]. Intra-rater agreement was based on a single rating, absolute agreement, 2-way mixed effects-model and inter-rater agreement on a single rating, absolute agreement, 2-way random-effects model [24]. In the benchmark scale used to evaluate ICC, values below 0.5 indicate poor agreement and those between 0.5 and 0.75, moderate agreement. We considered good ICC agreement values as between 0.75 and 0.9 and excellent agreement above 0.9 [24]. Finally, agreement between raters (by pairs of raters) was assessed by Bland–Altman plots; mean of differences or bias and limits of agreement were calculated [25]. P-values lower than 0.05 were considered as statistically significant. Statistical Package for the Social Sciences (SPSS) version 23.0 for Windows (IBM, Armonk, New York, United States of America) and Stata version 15 (StataCorp, College Station, Texas, United States of America) were used for statistical analysis.

### Results

All the participants were right-handed. Among the patients, no significant differences in muscle thickness of the dominant forearm were observed between men and women (mean difference 0.06 mm, 95%CI -3.03 to 3.15). Differences by sex could not be analyzed in the healthy volunteers, as only two men participated.

**Fig. 3:**
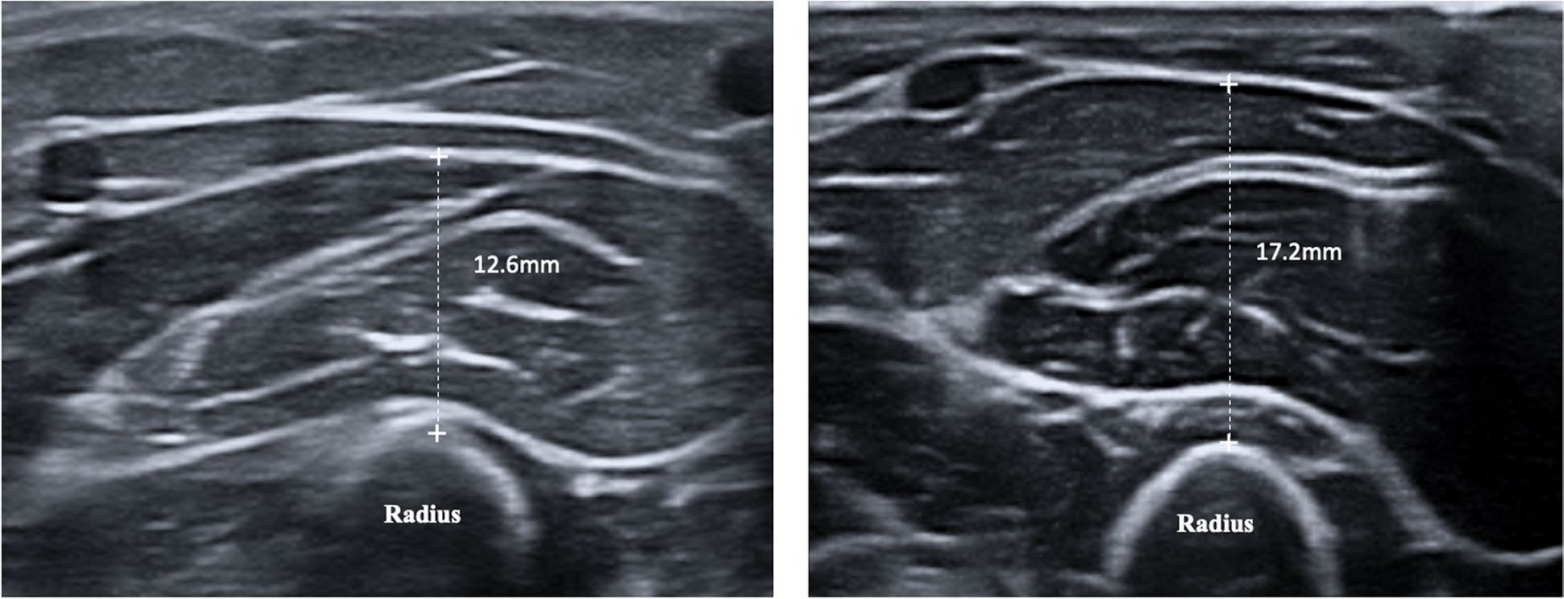
Forearm muscle thickness sonogram, Legend: Image obtained in a patient (left) and healthy volunteer (right), showing quantitative differences

Twenty-five trios of inter-rater measurements were evaluated, as described in Table 1. Mean muscle-thickness values were calculated for each participant according to the following formula: [mean A + mean B + mean C]/3. The results were 14.6 ± 2.0 mm in the older patients, and 18.4 ± 2.6 mm in the younger healthy volunteers (mean difference 4.4 mm [95%CI 2.4 mm to 6.3 mm], *p* < 0.001). Figure 3 shows obtained images in a patient and a healthy volunteer.

**Table 1:** The intra- and inter-rater agreement

	**Rater A**				**Rater B**				**Rater C**				
**Patients (> 70 y)**	**A1**	**A2**	**A3**	**Mean A**	**B1**	**B2**	**B3**	**Mean B**	**C1**	**C2**	**C3**	**Mean C**	**Mean ABC**
1	12.5	12.5	10.6	11.9	11.1	10.8	11.9	11.3	9.2	10.2	9.7	9.7	10.9
2	13.3	14.5	13.6	13.8	14.7	14.9	13.3	14.3	15.3	14.3	15.2	14.9	14.3
3	16.8	17.6	17.1	17.2	18.6	17.2	17.0	17.6	18.2	18.0	17.8	18	17.6
4	11.8	12.5	12.0	12.1	16.3	16.5	16.4	16.4	14.8	15.2	15.3	15.1	14.5
5	12.0	12.6	12.2	12.3	10.5	11.3	11.5	11.1	12.4	13.0	13.5	13.0	12.1
6	13.7	14.1	14.4	14.1	17.4	17.5	17.8	17.6	18.1	18.8	17.0	18.0	16.5
7	14.7	13.2	13.2	13.7	17.3	17.1	16.3	16.9	17.1	17.3	17.3	17.2	15.9
8	12.6	14.0	13.5	13.4	14.9	15.0	13.9	14.6	14.7	14.6	14.6	14.6	14.2
9	10.2	12.2	12.6	11.7	14.3	14.3	13.1	13.9	13.3	14.6	14.3	14.1	13.2
10	14.2	15.0	14.7	14.6	14.1	14.8	13.6	14.2	15.8	14.3	15.8	15.3	14.7
11	10.6	10.1	9.3	10.0	10.8	12.6	12.6	12.0	14.8	18.1	17.9	16.9	13.0
12	12.2	10.7	11.3	11.4	12.0	10.5	12.2	11.6	14.1	10.6	10.9	11.83	11.6
Volunteers (< 50 y)	A1	A2	A3	Mean A	B1	B2	B3	Mean B	C1	C2	C3	Mean C	Mean ABC
1	16.5	17.1	16.3	16.6	19.9	18.8	18.4	19.0	24.4	22.4	24.3	23.7	19.8
2	14.2	14.4	17.6	15.4	14.9	16.0	14.8	15.2	14.2	14.8	14.6	14.5	15.1
3	18.2	17.3	17.4	17.6	17.0	16.8	17.3	17.0	21.8	22.2	22.0	22.0	18.9
4	17.5	17.6	19.5	18.2	18.5	18.5	19.7	18.9	19.5	19.5	20.1	19.7	18.9
5	17.4	16.8	16.8	17.0	15.9	16.6	16.4	16.3	16.2	17.8	18.2	17.4	16.9
6	17.7	17.3	16.7	17.2	17.3	16.8	18.4	17.5	18.0	18.0	18.9	18.3	17.7
7	18.5	17.0	18.8	18.1	19.0	18.9	18.9	18.9	19.5	19.4	19.3	19.4	18.8
8	25.3	24.7	23.3	24.4	25.7	25.3	25.8	25.6	25.8	24.7	25.6	25.4	25.1
9	17.1	16.2	15.9	16.4	18.0	18.2	16.3	17.5	22.0	23.1	21.6	22.2	18.7
10	17.6	17.6	17.3	17.5	17.7	17.8	17.7	17.7	17.7	18.0	18.1	17.9	17.7
11	19.9	20.4	19.1	19.8	19.8	21.1	21.1	20.7	17.1	21.5	21.1	19.9	20.1
12	13.9	13.2	13.5	13.5	16.4	15.6	15.5	15.8	15.2	14.4	14.5	14.7	14.7
13	17.6	15.0	16.6	16.4	17.5	18.0	17.9	17.8	16.7	16.6	16.2	16.5	16.9

Legend: Muscle thickness of forearm in older patients and healthy volunteers, measured by three novice raters using ultrasound

The intra-rater agreement analysis showed excellent results: ICC for raters A, B, and C was 0.947 (95%CI 0.902 to 0.974), 0.969 (95%CI 0.942 to 0.985) and 0.950 (95%CI 0.907 to 0.975), respectively (Table 2). Comparison between all three raters showed good inter-rater agreement, with an ICC of 0.873 (95%CI 0.73 to 0.94). However, comparisons between raters had some disparities depending on the pair. Pairs A-B and B-C showed good agreement, with ICC of 0.89 (95%CI 0.97 to 0.96) and 0.9 (95%CI 0.78 to 0.96), respectively. When comparing A and C, the ICC was 0.83 (95%CI 0.43 to 0.94), with the lower limit of the confidence interval falling into the range of poor agreement.

**Table 2:** Inter- and intra-rater reliability of ultrasound muscle thickness measurements

	**Intraclass correlation coefficient**
Inter-rater reliability	0.873 (95% CI 0.73 to 0.94)
Intra-rater reliability	
-Rater A	0.947 (95% CI 0.902 to 0.974)
-Rater B	0.969 (95% CI 0.942 to 0.985)
-Rater C	0.950 (95% CI 0.907 to 0.975)

Legend: *95% CI* 95% confidence interval

**Fig. 4:**
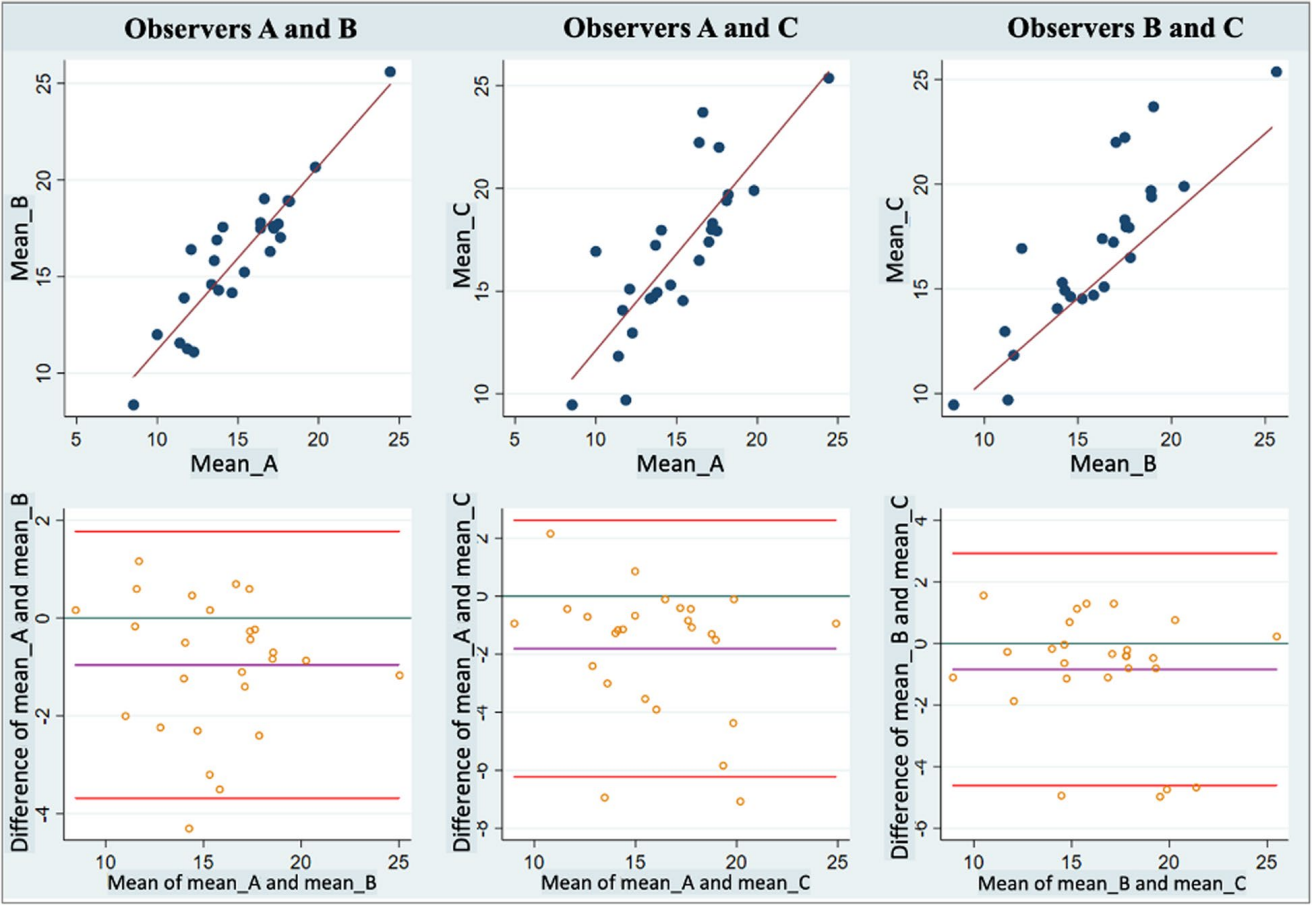
The Bland and Altman plots show the levels of bias and limits of agreement

Bland and Altman plots (Fig. 4**)** showed different levels of bias and limits of agreement, ranging from -0.52 in the B-C comparison to -1.37 in the A-C comparison. Moreover, pairs of measurement showed no patterns in the distribution of points in the plot area. From a total of 17 questions considered as relevant to assess feasibility, 4 answers were unknown or not as expected; these were specifically addressed by describing actions to overcome potential barriers to implementation (Table 3). Professionals from the Geriatrics and Rehabilitation Departments considered that information provided by muscle US could have clinical and prognostic implications. Although no infrastructure investment was required, the assessment could be considered time-consuming. The researchers in charge of examinations (1 geriatrician and 2 rehabilitation specialists) received a 45-min theory class followed by a practicum in which 5 supervised examinations were carried out.

**Table 3:** The TELOS components

Components	Questions to be considered	Expected answer	Reported answer	Actions to address potential barriers to implementation
Technology	Is the required equipment available in the institution?	Yes	Yes	–
	Will we need third-party resources?	No	No	–
	Is staff properly trained to implement the intervention or will new skills be needed?	Yes	No	45-min theory class + practicum (5 supervised examinations)
Economics	Are all the costs well-defined?	Yes	Yes	–
	Is the intervention expensive?	No	No	–
	Is the time–cost acceptable?	Yes	Yes	–
Legal requirements	Does the new intervention conflict with legal requirements?’	No	No	–
	Have we ensured that we are following all the standards of good clinical practice?	Yes	Yes	–
Operational needs	Are all of the tasks properly defined?	Yes	Yes	–
	Are the involved third parties willing to participate?	Yes	Yes	–
	Do new teams have to be established?	No	No	–
	Do we need to reorganize the processes?	No	No	–
	Will there be staff resistance to the change?	No	Unknown	Discuss the views and questions of all the parties involved
	Will there be training costs?	No	Yes	Low cost, incorporated into departmental budgeting
Scheduling	Given our current experience, is the intervention realistic?	Yes	Yes	–
	Are there any timescale pressures to be met?	No	No	–
	Will the intervention deliver meaningful benefits for patients?	Yes	Unknown	Incorporate a subproject to assess clinical and prognostic implications

Legend: Description of the technological, economic, legal, operational, and scheduling (TELOS) components and expected answers supporting the feasibility of introducing muscle ultrasound as a diagnostic procedure

The inclusion of US assessment in the geriatric ward fulfilled legal requirements. Unlike muscle US of lower limbs, for which patients must remove items of clothing, the mean total time spent in the examination room was only 12.5 min per assessment; the actual measurement was easily performed in less than 10 min per patient. No patients reported discomfort during the examination, and there were no adverse or unexpected effects except one patient who fell asleep during the procedure.

## Discussion

This pilot study showed US technology to be a reliable and feasible tool to measure muscle characteristics in older adults. Both quantity and quality are key measurements, as loss of muscle strength is essential for sarcopenia diagnosis. Our study focused on using researchers newly trained in US to assess muscle thickness in hospitalized geriatric patients who could maintain the protocol’s required sitting position. The good inter-rater and excellent intra-rater reliability data obtained after a 45-min training session demonstrated that US is an accessible tool for physicians interested in objective muscle assessment. The US is an inexpensive, non-invasive technique that uses no ionizing radiation. Modern equipment is portable, making it easy to use in clinical practice. To date, very few research groups have closely examined the usefulness of forearm US in the diagnosis of muscle diseases [26–29]; one of the groups found a close association between US muscle thickness and cross-sectional area measured by MRI in the forearm of young and middle-aged individuals [26]. In the present study, a significant forearm muscle thickness difference was observed between older patients and younger healthy volunteers; however, the reasons for these differences (age-related loss of muscle mass, physical inactivity, medications, comorbidities, etc.) offer an interesting and necessary question for future research.

A major drawback of US-based measurements is the lack of standardization with respect to aspects such as preferred muscle group, patient positioning, pretest exercise, dominant vs. non-dominant arm, or relaxed vs. contracted muscles. In our study, muscle thickness was measured at the proximal lateral forearm, 30% proximal of the distance between the styloid process of the radius and the insertion of the biceps brachii muscle into the radial tuberosity, which is the widest and most accessible part of the forearm in a neutral position.

Other US protocols exist for measuring the forearm musculature. These protocols generally evaluate the anterior part of the forearm at different levels, have usually sought to demonstrate the maximum handgrip strength [27], a good correlation between handgrip strength and muscle thickness [28], as an indicator of physical performance [29], and have shown good intra-rater and inter-rater reliability [4, 30–32]. However, there is no consensus recommending a specific measurement protocol for the forearm. Since lower arm muscles are more readily accessible than other more studied muscles and their measurement shows good reliability, this option for screening and diagnosis of muscle disorders could be very useful in clinical assessments, particularly in outpatient settings where time for examinations is very limited. In hospitalized patients who cannot maintain the sitting position, it would be better to assess other muscles (e.g., quadriceps in the supine decubitus position). However, in the forearm muscle thickness evaluation, the patient need only be seated with a bare forearm held steady in a supported position; this approach optimizes clinical time and resources in patients who can maintain this posture for approximately five minutes.

Our results suggest that the feasibility of the procedure is fairly good. Only a short training time was required, although there is a learning curve related to measuring distances and muscle area. Positioning the patient, handling the probe, and even the force applied by the examiner during the assessment are aspects that might influence the accuracy of the measurements.

The study of US feasibility in a geriatric setting may be considered a strong point of our research, as well as the use of three observers and a homogeneous population of older patients to support our reliability analysis. Limitations of the study include the relatively small group of participants, the unknowns that are inherent to the technique itself (as described above), and the lack of a test–retest analysis. Although the lack of a test–retest is a limitation of the design, the reliability obtained through the methodology (image capture and thickness measured) achieved the aims of our study. This study aimed to demonstrate how physicians with no previous experience in muscle assessment by US could perform muscle thickness assessment with a high degree of reliability after brief training. The methodology included training in four specific areas: (a) general US measurement techniques (correct grip and positioning of the US probe, amount of gel to be used, and attention to the pressure exerted on the tissues); b) anatomical location of the probe placement point on the forearm; (c) recognition of the anatomical structures in the ultrasound image obtained, and (d) correct measurement of structures in the US image captured.

## Conclusion

Our findings suggest that US is a feasible tool to assess thickness of forearm muscles with good inter-rater and excellent intra-rater reliability, requiring minimal training in the technique and limited time during the outpatient clinical examination. Therefore, it could be a suitable option in Geriatrics and Rehabilitation settings for the assessment of muscle characteristics and follow-up of patients with sarcopenia.

## Data Availability

The datasets used during the current study are available from the corresponding author upon reasonable request.

## Abbreviations

BAMS: The Belgian Ageing Muscle Society
BIA: Bio-Impedance Analysis
CI: Confidence Interval
DEXA: Dual-energy X-ray Absorptiometry
EGWSOP2: European Working Group of Sarcopenia on Older People 2
ESPEN: The European Society of Clinical Nutrition and Metabolism
EuGMS: The European Union Geriatric Medicine Society
ICC: Intraclass Correlation Coefficient
MRI: Magnetic Resonance Imaging
SARCUS: SARCopenia measurement by UltrasSound
SD: Standard Deviation
SPSS: Statistical Package for the Social Sciences
STROBE: Strengthening the Reporting of Observational Studies in Epidemiology
TELOS: Technological, Economic, Legal, Operational, and Scheduling components
US: Ultrasound
